# A New Insecticidal Sesquiterpene Ester from *Celastrus Angulatu*s

**DOI:** 10.3390/molecules14041396

**Published:** 2009-03-30

**Authors:** Shao-peng Wei, Zhi-qin Ji, Ji-wen Zhang

**Affiliations:** 1 Institute of Pesticide science, Northwest A&F University, Yangling, Shaanxi 712100, P.R. China; 2 College of Sciences, Northwest A&F University, Yangling, Shaanxi 712100, P.R. China; E-mails: weishaopeng8888@yahoo.com.cn (S-P.W); jizhiqin@nwsuaf.edu.cn (Z-Q.J)

**Keywords:** *Celastrus angulatus*, β-dihydroagarofuran sesquiterpene, Insecticidal activity

## Abstract

A new sesquiterpene polyol ester with a β-dihydroagarofuran skeleton, NW37 (**1**), and three known compounds NW13 (**2**), NW16 (**3**) and NW35 (**4**) were isolated by bioassay-guided fractionation from the highly polar MeOH extracts of the root bark of *Celastrus angulatus*. Their chemical structures were elucidated mainly by analyses of MS and NMR spectral data. The insecticidal activity of compound **1** against 4^th^ instar *Mythimna separata* larvae with a KD_50_ value of 252.3 μg·g^-1^ was demonstrated.

## 1. Introduction

Various β-dihydroagarofuran sesquiterpene polyol esters and pyridine alkaloids, some of which exhibit insect antifeedant, insecticidal, antitumor, reversing multidrug resistance, anti-HIV, and immunosuppressive activities, have been obtained from the plants of the Celastraceae family [1,2,3,4,5,6,7,8,9,10]. *Celastrus angulatus*, a plant of the this family, is widely distributed in China and used for the treatment of rheumatism in traditional Chinese medicine and as an insecticide [11,12]. In our previous studies, some antifeedant, narcotic, and insecticidal ingredients were isolated from the toluene extracts of the root bark of *C. angulatus*. To obtain a sufficient number of compounds for QSAR research on their insecticidal activity against *Mythimna separata*, the chemical constituents from the root bark of *C. angulatus* were re-investigated guided by activity-guided fractionation. These studies have led to the isolation of a novel sesquiterpene polyol ester NW37 (**1**). In this paper, the isolation, structure elucidation and insecticidal activity of compound **1** were presented.

## 2. Results and Discussion

Four sesquiterpene polyol esters **1**-**4** were isolated from the MeOH extracts of the root bark of *C. angulatus* by macroporous resin column chromatography and RP-HPLC, and their structures were elucidated on the basis of UV, HR-ESI-MS and NMR spectroscopic evidence. Compound **1**, a white powder, analyzed for C_38_H_52_O_14_ by HR-ESI-MS (*m/z* 750.3695 [M+NH_4_] ^+^, calculated 750.3700), and NMR spectra data (Table 1). Its IR spectrum revealed characteristic ester absorptions at 1,741 cm^-1^, and a free hydroxyl absorption at 3,510 cm^-1^. The UV spectrum contained an aromatic moiety (232 and 275 nm). The NMR spectra suggested the presence of three acetate esters, δ C 169.85 (CO), 169.60 (CO), 169.47 (CO), 21.59 (CH_3_), 21.28 (CH_3_), 20.53 (CH_3_), δ H 2.10 (3H, s), 2.07 (3H, s), 1.46 (3H, s), one benzoate ester, δ C 164.64 (CO), 133.96 (CH), 130.34 (2×CH), 128.60 (2×CH), 128.52 (C), δ H 8.00 (2H, d, *J*=7.0 Hz), 7.59 (1H, t, *J*=7.0 Hz), 7.45 (2H, t, *J*=7.0 Hz) and two α-methylbutanoate esters, δ C 176.68 (CO), 175.42 (CO), 41.28 (CH), 41.22 (CH), 26.65 (CH_2_), 26.58 (CH_2_),16.68 (CH_3_), 16.49 (CH_3_), 11.82 (CH_3_), 11.67 (CH_3_), δ H 2.59 (1H, m), 2.50 (1H, m), 1.80 (2H, m), 1.55 (2H, m), 1.25 (3H, d, *J*=2.0 Hz), 1.23 (3H, d, *J*=2.0 Hz), 0.96 (6H, m). The ^1^H-NMR of **1** showed the presence of three methyl groups at δ 1.49 (3H, s, H-13), 1.65 (3H, s, H-14), 1.62 (3H, s, H-15). Based on the published literature [13,14], the ^1^H-^1^H COSY spectrum signals at δ 5.62 (1H, d, *J*=3.5 Hz, H-1), 5.56 (1H, dd, *J*=3.5 Hz, 3.0, H-2), 6.25 (1H, s, H-6), 5.32 (1H, d, *J*=3.0 Hz, H-8)and 5.68 (1H, s, H-9) can be assigned to five protons attached to carbon atoms bearing secondary ester groups, while signals at δ 4.87 (1H, d, *J*=10.0 Hz, H-12a) and δ 4.83 (1H, d , *J*=10.0Hz, H-12b) can be assigned to the two protons attached to carbon atoms bearing primary ester groups. 

The ^13^C-NMR (DEPT) spectrum of the parent skeleton of **1** showed three methyls at δ 24.72, 25.83 and 29.76, one methylene at δ 42.27, one methylene attached to an oxygen function at δ 65.72, one methine at δ 53.35, five methines attached to an oxygen function at δ 71.09, 68.26, 75.58, 76.32 and 72.31, one quaternary carbon at δ 54.20, and three quaternary carbons attached to an oxygen function at δ 70.06, 83.69 and 91.64, whose chemical shifts were very similar to those of reported β-dihydro-agarofurans. It was thus determined that compound **1** was a β-dihydroagarofuran sesquiterpene substituted with three acetate, one benzoate and two α-methylbutanoate esters. The ester group distributions were determined from the HMBC spectrum, which showed cross-peaks between H-9 and the carbonyl at δ 164.64 of the benzoate ester, H-12, H-8 and the carbonyl at δ 176.68, 175.42 of the two α-methylbutanoate esters, H-1, H-2, H-6 and the carbonyls at δ 169.85, 169.60, 169.47 of three acetate esters, respectively. In the molecular skeleton of β-dihydroagarofuran sesquiterpenes, H-1 and H-6 have axial stereochemistry. From the results of the NOESY spectrum of **1**, the correlation between H-6 and H-9 indicated the presence of H-9_eq_ and the correlation between H-14 and H-8 indicated the presence of H-8_eq_ (Figure 2).Therefore, compound **1** was identified as 1β,2β,6α-triacetoxy-8β,12-di-(α-methyl)butanoyl-9α-benzoyloxy-4α-hydroxy-β-dihydroagarofuran.

**Figure 1:**
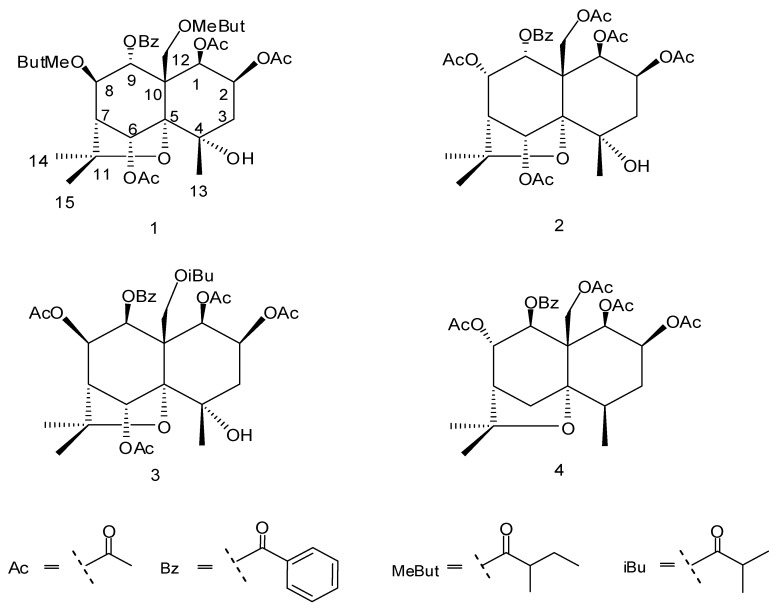
The structures of compounds 1-4.

NW13(**2**), NW16(**3**) and NW35(**4**) were known compounds, and there were characterized as 1β,2β,6α,8α,12-pentaacetoxy-9α-benzoyloxy-4α-hydroxy-β-dihydroagarofuran (**2**) [13], 1β,2β,6α,8β-tetraacetoxy-9β-benzoyloxy-12-isobutanoyloxy-4α-hydroxy-β-dihydroagarofuran (**3**)[14] and 1β,2β,8α,12-tetraacetoxy-9β-benzoyloxy-β-dihydroagarofuran (Angulatueoid B, **4**) [15] on the basis of UV, IR, ^1^H- and ^13^C-NMR spectroscopic evidence.

**Table 1:** The NMR data of compound **1**. (CDCl_3,_^1^H-NMR at 500 MHz, ^13^C-NMR at 125MHz, respectively)

No.	Δ C (DEPT)	δH (*J*, Hz)	HMBC
1	71.09 CH	5.62 (1H, d, *J*=3.5 Hz)	C-2,C-10, C=O of Ac
2	68.26 CH	5.56 (1H, dd, *J*=3.5 Hz, *J*=3.0 Hz)	C-10, C=O of Ac
3	42.27 CH_2_	2.24 (1H, m), 2.00 (1H, m)	C-1,C-2,C-4,C-5,C-13
4	70.06 C		
5	91.64 C		
6	75.58 CH	6.25 (1H,s)	C-5,C-7,C-8,C-10,C-11, C=O of Ac
7	53.35 CH	2.37 (1H, d, *J*=3.0 Hz)	C-5,C-6,C-8,C-9
8	76.32 CH	5.32 (1H, d, *J*=3.0 Hz)	C=O of MeBut
9	72.31 CH	5.68 (1H, s)	C-5,C-7,C-8,C-10,C-12, C=O of Bz
10	54.20 C		
11	83.69 C		
12	65.72 CH_2_	4.87 (1H,d, *J*=10.0 Hz) 4.83 (1H,d, *J*=10.0 Hz)	C-1,C-5,C-9,C-10, C=O of MeBut
13	24.72 CH_3_	1.49 (3H, s)	C-4, C-5
14	25.83 CH_3_	1.65 (3H, s)	C-7, C-11
15	29.76 CH_3_	1.62 (3H, s)	C-7, C-11
Ac	169.85 (CO), 21.59 (CH_3_)	2.10 (3H, s)	
Ac	169.60 (CO), 21.28 (CH_3_)	2.07 (3H, s)	
Ac	169.47 (CO), 20.53 (CH_3_)	1.46 (3H, s)	
MeBut	176.68 (CO) 41.28 (CH), 26.65 (CH_2_), 11.82 (CH_3_), 16.68 (CH_3_)	2.59 (1H, m), 1.80 (2H, m), 1.25 (3H, d, *J*=2.0 Hz), 0.96 (3H, m)	
MeBut	175.42 (CO) 41.22 (CH), 26.58 (CH_2_), 11.67 (CH_3_), 16.49 (CH_3_)	2.50 (1H, m), 1.55 (2H, m), 1.23 (3H, d, *J*=2.0 Hz), 0.96 (3H, m)	
Bz	164.64 (CO), 133.96 (CH), 130.34 (2×CH), 128.60 (2×CH), 128.52 (C)	8.00 (2H, d, *J*=7.0 Hz), 7.59 (1H, t, *J*=7.0 Hz), 7.45 (2H, t, *J*=7.0 Hz)	

**Figure 3:**
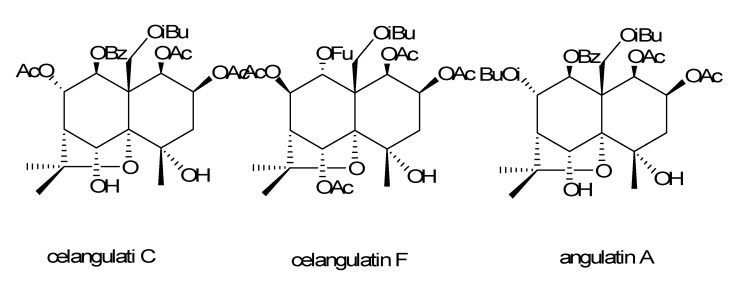
The structures of celangulatin C, celangulatin F and angulatin A.

The insecticidal activities of compounds **1**-**4** against 4^th^ instar larvae of *Mythimna separata* were tested by the leaf disc method (for thr methodology see [13,14,16,17]). The result showed that the KD_50_ value for compound **1** was 252.3 μg· g^-1^. The symptoms displayed by the *Mythimna separata* indicated that these compounds have stronger insecticidal but not narcotic or antifeedant activities. On comparison of the KD_50_ data of compounds **1**-**4** presented in Table 2 and other compounds isolated in our laboratory, such as celangulatin C (KD_50_=280.4 μg· g^-^1), celangulatin F (KD_50_=201.5 μg· g^-1^) and angulatin A (KD_50_=300.9 μg· g^-1^) (for structures see Figure 3 ) [18], it was very interesting to note that compound **4** exhibited weaker activities than compound **1**-**3** and other compounds. For the structure of these compounds, it is obvious that the stereochemistry and the type of the ester groups at C-1 and C-2 in these compounds are similar, and the differences between them are the substitution groups at C-8, C-9 and C-12. In addition, the protons of C-4 and C-6 of compound **4** were not substituted by hydroxyl or ester groups, which indicated that the C-4 and C-6 substituents have a positive effect on the insecticidal activity. Moreover, these results suggested that the substitutes and stereochemistry of C-8, C-9, and C-12 play important roles in these compounds [13,18,19,20].

**Table 2:** The KD_50_ data of **1**~**4** and other compounds.

Compounds	KD_50_ (μg· g^-1^)
**1**	252.3
**2**	290.1
**3**	360.2
**4**	884.3
Celangulatin C	280.4
Celangulatin F	201.5
Angulatin A	300.9

## 3. Experimental

### 3.1. General

Melting points were measured on a Yanagimoto apparatus and are uncorrected. Optical rotations were measured on a Perkin-Elmer 341 polarimeter (USA). IR spectra were determined on an IR-450 instrument (KBr plate). ^1^H-NMR, ^1-^C-NMR, DEPT, COSY, HMQC, HMBC, and NOESY spectra were recorded on Bruker Avance 500 MHz NMR Spectrometer with CDCl_3_ as solvent and TMS as internal standard. HR-ESI-MS was obtained on a Bruker Apex II mass spectrometer. Finnigan LCQ Advantage MAX LC/MS, equiped with Surveyor DAD detector and Hypersil ODS_2_ C_18_ column (4.6×250 mm, 5 μm, Dalian Elite Analytical Instruments Co., Ltd., P.R. China), was used to analyse the samples. Compounds were purified with a Waters 600E HPLC apparatus equipped with a Hypersil ODS_2_ C_18_ preparative column (20 × 250 mm, 10 μm, Dalian Elite Analytical Instruments Co., Ltd., P.R. China) , MeOH-H_2_O (55: 45) as eluent, UV detector set at 230 nm.

### 3.2. Plant material

The root bark of *C. angulatus* was collected in Qinling mountain, Taibai County, Shaanxi Province, People’s Republic of China, in October 2007, authenticated by Dr. Hua Yi of the College of Life Sciences, Northwest Agricultural & Forestry University, and dried in the shade (at room temperature). Voucher specimens (samples no. NWAU2007-A18) were deposited at the College of Plant Protection, Northwest Agricultural & Forestry University.

### 3.3. Extraction and isolation

The dried and pulverized root bark (2.0 kg) of *C. angulatus* was extracted four times with MeOH (6.0L) under reflux. The extracted material (120 g) was adsorbed in a D101 macroporous resin (Hebei Cangzhou Chemical Co., Ltd., P.R. China) column (5.0×150 cm) and eluted with MeOH-H_2_O (5:5, 6:4, 7:3), and 100 fractions of ca. 500 mL each were collected. After removal of the solvents under reduced pressure, fractions were analysed by LC/DAD/MS, and similar ones were combined. The insecticidal activity of every fraction was assayed. Then the fractions which containing unknown sesquiterpene polyol esters were selected for further purification by RP-HPLC column, affording four compounds: NW37 (**1**, 75 mg), NW13 (**2**, 78 mg), NW16 (**3**, 92mg) and NW35 (**4**, 35mg).

**Figure 2:**
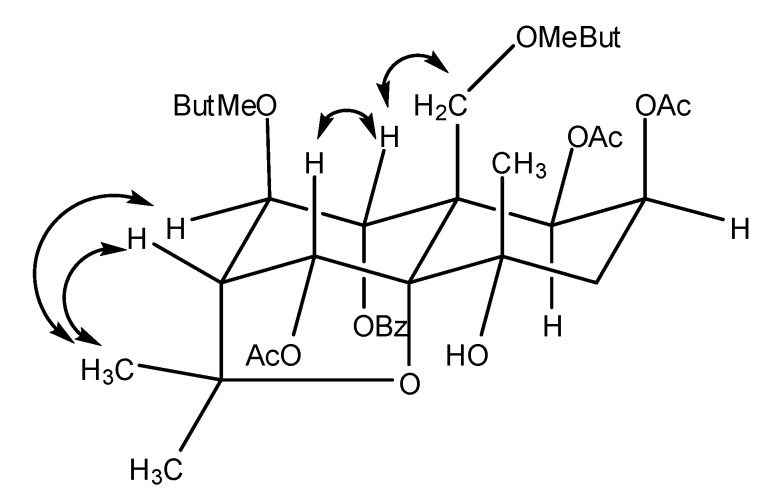
Major NOESY correlations in **1**.

*Compound* **1**: C_38_H_52_O_14_, white powder, -12.0° (CH_3_COCH_3_, c 1.20); IR *v*: 3510,2926, 1741, 1632, 1380, 1232, 1060,891, 712 cm^-1^; UV: 232, 275 nm; ESI-MS (MS/MS): *m/z* (%) 755 [M+Na] ^+^ (17), 695 [M+Na-AcOH] ^+^ (80), 653 [M+Na-MeBuOH] ^+^ (100), 633[M+Na-BzOH]^+^ (21), 593 [M+Na-AcOH-MeBuOH] ^+^(12). ^1^H- and ^13^C-NMR (CDC_l3_) see Table 1. Major NOESY correlations Figure 2.

### 3.4. Insecticidal activity

Toxic leaf discs of known area were treated with known amounts of the test samples dissolved in acetone (acetone and celangulin V were used as negative and positive control). The 4^th^ instar larvae of *M. separata* were fed with the discs for 12 h (repeated 10 times for each sample). After 24 h, the numbers of knocked-down larvae (symptoms: the larvae were narcotized and could not move; the bodies were immobilized and very soft; and the response disappeared completely) were recorded, and the toxicity was ascertained by estimating the median knock-down dose (KD_50_ value) of the test sample [14].
